# Supplementary material for: Metabolic Syndrome Severity Score, Comparable to Serum Creatinine, Could Predict the Occurrence of End-Stage Kidney Disease in Patients with Antineutrophil Cytoplasmic Antibody-Associated Vasculitis
Source: J Clin Med. 2021 Dec 8;10(24):5744. doi: 10.3390/jcm10245744 (PMC8708376; doi:10.3390/jcm10245744)
Supplement: Supplementary file 1 [file jcm-10-05744-s001.zip › jcm-1500961-supplementary.pdf]

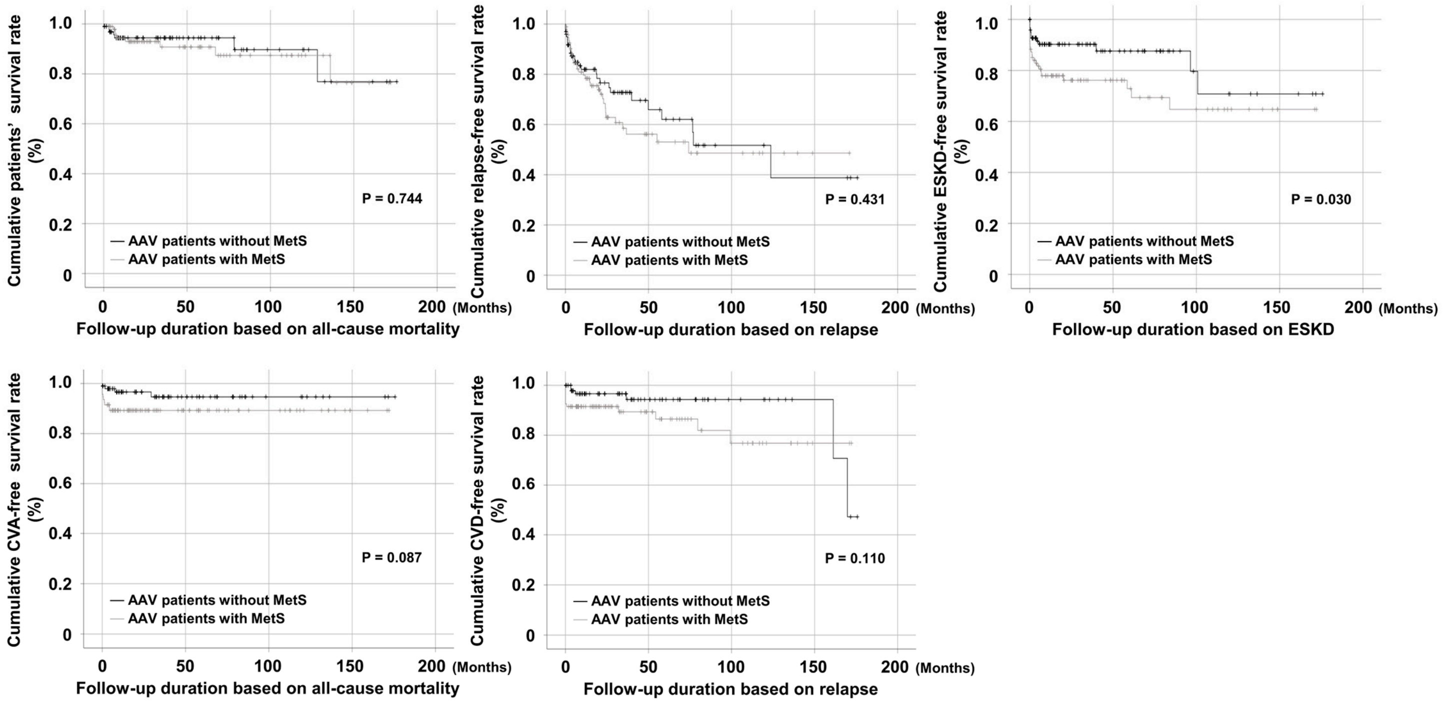

Figure S1. Comparison of the cumulative each poor outcome-free survival rate between AAV patients with MetS and those without.

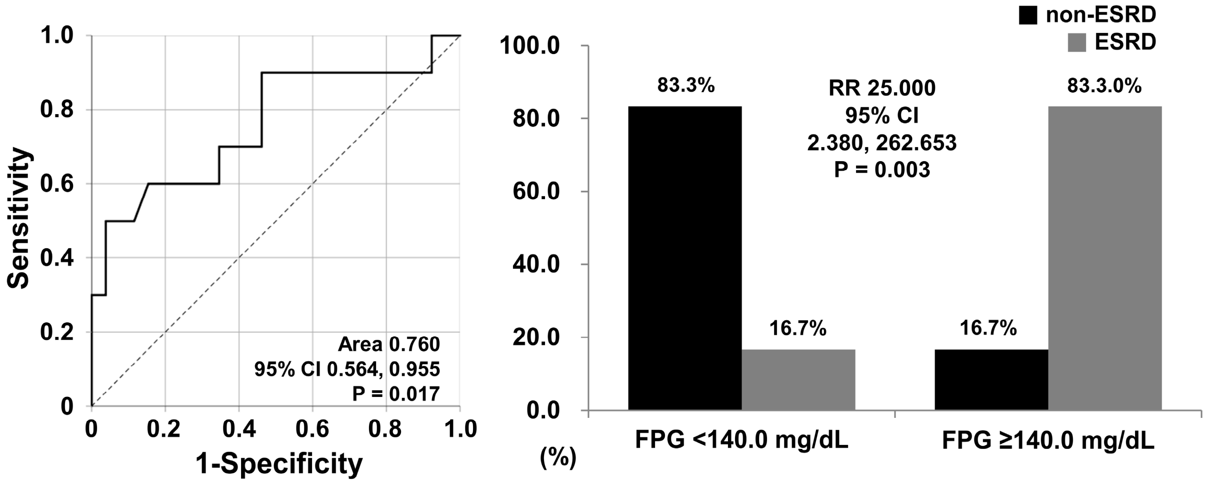

Figure S2. Comparison of the frequency of ESKD between AAV patients with FPG ≥140.0 mg/dL and those with FPG < 140.0 mg/dL.

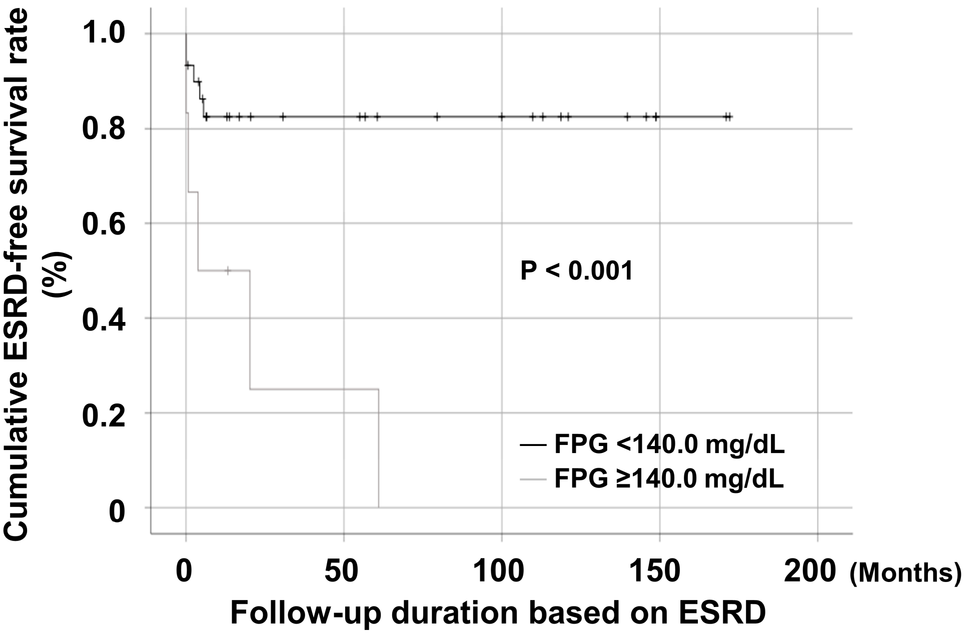

Figure S3. Comparison of the cumulative ESKD-free survival rate between FPG ≥ 140.0 mg/dL and those with FPG < 140.0 mg/dL.

Table S1. Correlation of MSSS with continuous variables at AAV diagnosis with MetS.

Variables	Correlation Coefficient	*p*-Value
Age (years)	0.340	0.042
Body mass index (kg/m^2)	-0.013	0.942
BVAS	-0.098	0.569
FFS	0.121	0.482
White blood cell count (/mm^3)	-0.213	0.213
Haemoglobin (g/dL)	-0238	0.163
Platelet count (x1,000/mm^3)	0.090	0.603
Blood urea nitrogen (mg/dL)	-0.077	0.654
Serum creatinine (mg/dL)	-0.019	0.911
Serum albumin (g/dL)	-0.363	0.030
ESR (mm/hr)	0.233	0.179
CRP (mg/L)	0.032	0.854

MSSS, metabolic syndrome severity score; AAV, ANCA-associated vasculitis; ANCA, antineutrophil cytoplasmic antibody; MetS, metabolic syndrome; BVAS, Birmingham vasculitis activity score; FFS, five-factor score; ESR, erythrocyte sedimentation rate; CRP, C-reactive protein.

Table S2. Comparison of poor outcomes and medications administered during follow-up between patients with MSSS ≥ 1.72 and those with MSSS < 1.72.

Variables	Patients with MSSS < 1.72 (*n* = 28)	Patients with MSSS ≥ 1.72 (*n* = 8)	*p*-Value
**Poor outcomes and follow-up duration**			
All-cause mortality	1 (3.6)	0 (0)	1.000
Relapse	15 (53.6)	5 (62.5)	0.709
ESRD	4 (14.3)	6 (75.0)	0.002
CVA	2 (7.1)	0 (0)	1.000
CVD	3 (10.7)	2 (25.0)	0.305

**Medications administered during follow-up**			
Glucocorticoid	28 (100)	8 (100)	N/A
Cyclophosphamide	16 (57.1)	4 (50.0)	1.000
Rituximab	6 (21.4)	2 (25.0)	1.000
Mycophenolate mofetil	6 (21.4)	1 (12.5)	1.000
Azathioprine	14 (50.0)	3 (37.5)	0.695
Tacrolimus	0 (0)	1 (12.5)	0.222
Methotrexate	1 (3.6)	1 (12.5)	0.400

Values are expressed as a median (interquartile range, IQR) or *n* (%). AAV, ANCA-associated vasculitis; ANCA, antineutrophil cytoplasmic antibody; MSSS, metabolic syndrome severity score; ESRD, end-stage renal disease; CVA, cerebrovascular accident; CVD, cardiovascular disease; N/A, not applicable.

Table S3. Cox hazards model analysis of variables at the time of AAV diagnosis for ESKD occurrence during follow-up in AAV patients (FPG rather than MSSS).

**Variables**	**Univariable**			**Multivariable**		
	**HR**	**95% CI**	***p* Value**	**HR**	**95% CI**	***p* Value**
Age	1.019	0.944, 1.099	0.635			
Male sex	0.932	0.293, 3.638	0.920			
Body mass index	0.779	0.624, 0.974	0.028			
MPA	2.266	0.585, 8.771	0.236			
GPA	2.090	0.533, 8192	0.290			
EGPA	0.027	0.000, 7.064	0.203			
MPO-ANCA (or P-ANCA) positivity	4.810	0.607, 38.140	0.137			
PR3-ANCA (or C-ANCA) positivity	0.675	0.086, 5.334	0.710			
BVAS	1.114	1.017, 1.221	0.020	1.100	0.901, 1.342	0.350
FFS	2.726	1.309, 5.677	0.007	1.394	0.334, 5.817	0.649
White blood cell count	1.000	1.000, 1.000	0.980			
Haemoglobin	0.665	0.471, 0.940	0.021	0.993	0.642, 1.535	0.975
Platelet count	0.988	0.992, 1.004	0.998			
Blood urea nitrogen	1.023	1.010, 1.035	<0.001	0.986	0.953, 1.019	0.399
Serum creatinine	2.508	1.604, 3.919	<0.001	2.778	1.473, 5.236	0.002
Serum albumin	0.555	0.198, 1555	0.263	1.471	0.125, 17.325	0.759
ESR	1.019	0998, 1.040	0.074			
CRP	1.006	0.997, 1.015	0.209			
FPG	1.009	1.001, 1.018	0.029	1.014	1.000, 1.029	0.049

AAV, ANCA-associated vasculitis; ANCA, antineutrophil cytoplasmic antibody; ESRD, end-stage renal disease; FPG, fasting plasma glucose; MPA, microscopic polyangiitis; GPA, granulomatosis with polyangiitis; EGPA, eosinophilic GPA; MPO, myeloperoxidase; P, perinuclear; PR3, proteinase 3; C, cytoplasmic; BVAS, Birmingham vasculitis activity score; FFS, five-factor score; ESR, erythrocyte sedimentation rate; CRP, C-reactive protein; MSSS, metabolic syndrome severity score.
